# Research progress of metabonomics of blood endogenous small molecules in depression

**DOI:** 10.1192/j.eurpsy.2021.2055

**Published:** 2021-08-13

**Authors:** Y. Zhang, J. Ding, X. Cui

**Affiliations:** Pharmacy Lab, Xi’an Mental Health Center, Xi’an, China

**Keywords:** metabonomics, blood endogenous small molecules, Depression

## Abstract

**Introduction:**

Depression (MDD) is a serious mental illness, which greatly affects the quality of life of patients. Nowadays, the clinical diagnosis of MDD lacks sufficient objective basis, and the effect of drug treatment is unsatisfactory. Therefore, biomarkers are very important for the risk prediction, classification, diagnosis and prognosis of MDD.

**Objectives:**

Research progress of metabonomics of blood endogenous small molecules in depression

**Methods:**

Metabonomics is a newly developed discipline after genomics and proteomics, and is an important part of system biology. Metabonomics provides a new approach to explore the etiology, mechanism, prognosis and screening potential biomarkers of MDD. Blood contains almost all the small molecule metabolites in the body. The changes of metabolites in blood can represent the changes of metabolites in other body fluids. Moreover, this sample is easy to obtain and has less trauma, so it is the most common biological sample in clinical detection.

**Results:**

At present, there are many studies on the metabonomics of endogenous small molecules in MDD blood, which provides the possibility for further screening of MDD related biomarkers.

**Conclusions:**

In this paper, the research progress of related biomarkers in MDD blood is reviewed.

**Disclosure:**

No significant relationships.

